# Modification of Visual Contrast in the Dining Environment and Its Impact on Dietary Intake in Older Adults—A Systematic Review

**DOI:** 10.3390/nu18142338

**Published:** 2026-07-16

**Authors:** Molly Gaffney, Maeve Ryan, Tobias Loetscher, Ben Singh, Karen J. Murphy

**Affiliations:** 1 Trinity College, The University of Dublin, College Green, D02 PN40 Dublin, Ireland; mollygaffney18@gmail.com (M.G.); maeve.ni.riain@gmail.com (M.R.); 2 Technological University Dublin, Grangegorman Campus, 191 North Circular Road, D07 EWV4 Dublin, Ireland; 3 Behavioural & Social Sciences, Adelaide University, Adelaide, SA 5001, Australia; tobias.loetscher@adelaide.edu.au; 4 Alliance for Research in Exercise Nutrition and Activity (ARENA), Adelaide University, Adelaide, SA 5001, Australia; ben.singh@adelaide.edu.au; 5 Allied Health & Human Performance, Adelaide University, Adelaide, SA 5001, Australia

**Keywords:** visual contrast sensitivity, older age, dietary intake, weight loss, malnutrition, tableware, dementia, Alzheimer’s Disease

## Abstract

**Background/Objectives:** Malnutrition among older adults, particularly those in aged care, is a major contributor to morbidity and healthcare costs. Reduced visual contrast sensitivity, common with ageing and dementia, may impair the ability to distinguish food from tableware, potentially leading to decreased intake. This systematic review examined whether enhancing visual contrast in the dining environment improves dietary intake in older adults. **Methods:** Following PRISMA guidelines, five databases were searched from inception to March 2025. Studies were eligible if they involved adults aged ≥65 years and used visual contrast interventions (e.g., coloured tableware, lighting adjustments) aimed at improving food or fluid intake. A narrative synthesis was conducted, and study quality was assessed using the Mixed Methods Appraisal Tool. **Results:** Of 2901 records screened, four studies (five reports) met the inclusion criteria, involving a total of 64 participants. All studies implemented visual contrast enhancements, including high-contrast dishware and environmental modifications. Most reported increases in food or liquid intake, though statistical significance varied. Some studies also evaluated mealtime behaviours, functional abilities, and food waste, with mixed findings. Methodological limitations, including small sample sizes, short interventions, and inconsistent reporting limited the strength of evidence. **Conclusions:** Limited and low-quality evidence suggests a possible effect of modifying the dining environment to improve visual contrast for enhancing dietary intake in older adults, with visual contrast sensitivity, particularly those with dementia. However, the evidence gap is large and remains inconclusive. Future well-powered trials with standardised interventions and outcome measures are needed to determine whether these strategies can meaningfully reduce malnutrition in aged care settings.

## 1. Introduction

The impact of unintentional weight loss and malnutrition is widely reported in the literature, with significant associated health outcomes such as increased risk of infection, falls, fractures, more frequent/prolonged hospital admissions, and, ultimately, increased morbidity and mortality [1,2,3,4,5,6,7,8]. Older adults are especially vulnerable to malnutrition [9], with a 2019 systematic review and meta-analysis of 196 studies demonstrating that 23% of older adults in Europe are at high risk [10]. Malnutrition is particularly prominent among residents in aged care facilities [11,12]. A 2024 prevalence study involving 10 aged care homes in Australia found that 40% of participants were malnourished [13]. Furthermore, it is estimated that the annual financial burden of malnutrition in Australian nursing homes is approximately AU$9 billion [14]. Given the global ageing population is expected to triple by 2050 [15], the prevalence and financial burden of malnutrition will undoubtedly rise [16].

An environmental factor that may impact malnutrition is visual contrast, particularly in the dining environment [17,18,19]. Ageing is associated with a natural decline in various aspects of vision [20]. The process involves yellowing and loss of elasticity of the eye lens, as well as a decrease in reaction time and pupil size [21]. This decline in vision reduces visual contrast sensitivity, making it more difficult for older persons to decipher between different colours [22]. According to ERCO Lighting, visual contrast is “the visual process of perceiving the difference in brightness or colour between two objects or between an object and its surroundings” [23]. This refers to the ability to distinguish objects from their backgrounds. The effect of reduced visual contrast sensitivity is not only seen with older age but also emphasised in those with cognitive impairments, such as Alzheimer’s Disease (AD) or dementia [24,25,26,27]. The inability to see or distinguish food from a plate (e.g., white rice from a white plate) or liquid from a cup (e.g., white milk from a white cup) may contribute to reduced food and beverage intake [17,18,19], possibly leading to energy and nutrient deficiencies, weight loss, and malnutrition. Modifying the dining environment by increasing the contrast between food/beverages and tableware may assist those with reduced visual contrast by improving dietary intake and overall wellbeing, particularly in aged care facilities [17,18,28,29].

There are numerous studies focusing on nutritional status in older adults [30,31,32]; however, there is minimal research on exploring the impact of visual contrast sensitivity on dietary intake [17,18,28]. The studies available are limited by small sample sizes, varying eligibility criteria, methodological differences and conflicting results [17,18,28,29,33]. This systematic review aims to evaluate whether modification of visual contrast in the dining environment improves dietary intake for older adults, particularly those in aged care facilities.

## 2. Materials and Methods

### 2.1. Registration and Protocol

The protocol for this systematic review was prospectively registered in the PROSPERO database (ID: CRD420250651134). The results are reported in accordance with the PRISMA statement [34].

### 2.2. Databases and Search Strategy

A comprehensive electronic database search was undertaken using subject headings, keywords, and MeSH term searches for “aged,” “aged care,” “Alzheimer’s Disease,” “dementia,” “visual contrast,” “colour,” “dishware,” “meals,” “dietary intake,” “nutrition,” “weight loss,” and “malnutrition.” Boolean operators (AND, OR) were employed. No filters were applied. Supplementary Table S1 shows the MEDLINE search strategy and terms. Five databases were searched from inception to March 2025: MEDLINE, CINAHL (EBSCOhost), Embase, Emcare, and Scopus. Forward and backward citation chasing of each included article was conducted.

### 2.3. Eligibility Criteria

The eligibility criteria were developed using the population, intervention, comparison, outcomes, and study type (PICOS) [35] framework as follows: *Population*—Older adults (aged ≥ 65 years) residing in aged care facilities, acute settings, or the community, including clinical populations such as those with dementia and Alzheimer’s Disease. *Intervention*—Tableware with increased visual contrast, which may involve modifications to dishware, tablecloths, and lighting. The following definition of visual contrast was used: “the visual process of perceiving the difference in brightness or colour between two objects or between an object and its surroundings” [23]. Studies were excluded if they analysed modifications to the physical environment without addressing visual contrast, as they did not answer the specific research question of this review. *Comparison*—Studies with and without a comparator or control group were eligible to identify a broader range of evidence [36]. *Outcome*—The primary outcomes of interest were increased food and/or drink consumption and reduced risk, prevalence, or incidence of weight loss or malnutrition. These are pertinent to the research aim. Secondary outcomes include decreased food waste, a reduction in eating challenges, and less assistance required with eating. However, these were less commonly reported. *Study type*—Peer-reviewed experimental studies, including randomised controlled trials (RCTs), pre–post designs, crossover trials, quasi-experimental studies, and observational studies were eligible for inclusion. Case studies, conference abstracts, and dissertations were excluded, as they may omit important information and generally have a less rigorous peer-review process [37,38,39,40]. Only studies published in English were considered due to practical reasons such as feasibility and resource availability [41]. This may introduce language bias, which could pose a limitation [42,43,44]. However, recent research indicates that the exclusion of non-English studies has minimal effects on conclusions [45,46]. There was no restriction on publication timeframe. This allows for a more comprehensive collection of relevant studies, identifying both recent and older research [42,47]. This is particularly important to ensure a thorough review, given the limited evidence on this topic [17,18,28,29].

### 2.4. Screening Procedure

Search results were imported into Endnote X9 and then exported into Covidence software (Veritas Health Innovation, Melbourne, Australia) where each individual database was screened for potential studies by examining the titles and abstracts of references retrieved. Articles that were considered potentially eligible based on titles or abstracts were retrieved as full texts and screened against the inclusion criteria by two independent reviewers (M.R. and M.G.). Discrepancies were resolved by discussion with the involvement of a third reviewer (K.J.M.) where necessary. Duplicate papers were double-checked and excluded.

### 2.5. Data Extraction

Data were extracted independently by two reviewers (M.R. and M.G.) using a standardised data extraction form of review characteristics (e.g., number of included studies, study designs, number of participants, sample characteristics), intervention details (including adherence and compliance data), outcome measures, and overall results for any relevant outcomes.

### 2.6. Data Synthesis and Quality Appraisal

Synthesis of data was conducted independently in duplicate, using a narrative approach with a descriptive synopsis of data extracted. The results of individual studies were tabulated to provide a summary of the important data. Table 1 includes information on study design, sample size, participants, interventions, outcomes, and the main findings. Where possible, findings were compared across studies to identify patterns, similarities, and differences. The synthesis focused on the overall direction and consistency of the evidence, as well as the quality of the included studies. This approach allowed for a comprehensive summary of the evidence while considering the heterogeneity of study designs and outcomes. The Mixed Methods Appraisal Tool (MMAT) was applied to included studies to evaluate their methodological quality and assess risk of bias [48]. The tool was used by two independent reviewers (M.G. and M.R.), and discrepancies were resolved through discussion between authors. Studies were first classified by design. Then, the MMAT evaluated the quality of studies using five criteria for each study design. Qualitative studies were assessed based on the following criterion of design, sampling, and data collection/analysis methods; quantitative studies were evaluated for design clarity, sampling, and data analysis rigour; and mixed methods studies were assessed for the integration of qualitative and quantitative data. Each study was rated as meeting (“yes”), not meeting (“no”), or “can’t tell” for each criterion. A “yes” response imposed a 20% score, while a “no” or “can’t tell” response was assigned 0%, resulting in a maximum of 100%, with higher scores indicating better methodological quality.

## 3. Results

### 3.1. Study Selection

**Figure 1:**
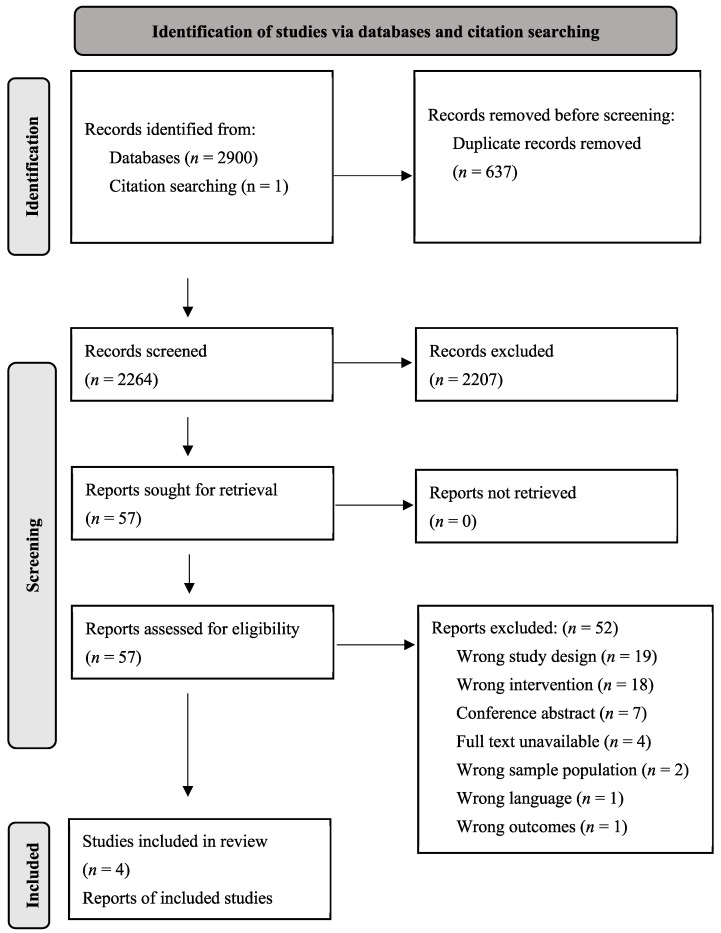
PRISMA flow diagram.

The electronic searches across the databases revealed 2901 papers of possible interest (see Figure 1 for PRISMA flow diagram). A total of 637 duplicates were removed, four full texts were unavailable, and 53 full-text studies were retrieved after title and abstract screening. Four studies [17,18,28,29] met the inclusion criteria, comprising five reports as one study [29] had two publications on different outcomes [29,33]. Reasons for exclusion can be seen in Figure 1. A meta-analysis was not conducted due to the heterogeneity of studies and outcome measures.

### 3.2. Study Characteristics

Table 1 details the characteristics of included studies [17,18,28,29,33]. All studies were conducted and reported within the last 25 years, with the most recent study published in 2020. The studies consisted of mixed methods (*n* = 1) and non-randomised quantitative designs (*n* = 4). Study timeframes and intervention periods were not consistently reported across all studies, with the longest test period lasting 30 days.

### 3.3. Population Characteristics

A total of 64 participants were included across the studies; sample sizes were small, ranging from 9 to 25. The majority were female (63%) and the remainder male (37%). Most studies included participants aged 65 years or older (range: 65–93 years), while one study specified all participants were aged 70 or above [17]. Studies were conducted in various long-term care facilities, including residential care, nursing home units, memory care units and assisted living facilities across Canada (*n* = 1), the United States (*n* = 2), and Norway (*n* = 1). All but one of the studies included participants with a confirmed diagnosis of AD or dementia, while the remaining study included participants with a probable diagnosis of AD [18]. Different tools were used across different sites to assess cognition, including the Montreal Cognitive Assessment (MoCA), Mini-Mental State Exam (MMSE), Minimum Data Set for Nursing Home Resident Assessment and Care Screening (MDS). The MDS is not a specific cognitive assessment tool; however, it can provide insightful population descriptors. Additionally, participants were required to be able to independently self-feed or require minimal assistance at mealtimes.

### 3.4. Quality Appraisal

An overview of the MMAT assessment for each report is provided in Table 2. The studies displayed various degrees of stringency, and MMAT scores ranged from 40 to 60%. The mixed methods study indicated slightly higher methodological quality than the non-randomised quantitative designs. While most papers consistently applied their interventions and addressed their research questions, several common limitations affect the overall generalisability and reliability of results. All studies were limited by their small sample size and unclear sampling methods. Compliance with the intervention was not explicitly stated in any of the studies. Studies failed to account for or report on controlling confounders and overall outcome data. In general, the standard of reporting was poor, making it challenging to assess methodological quality.

### 3.5. Interventions

All interventions focused on modifying the dining environment through contrast enhancements to improve dietary intakes among aged care residents. Dunne et al. [18] used high-contrast red tableware to provide maximal contrast to the food served during the testing period for the initial study. In a follow-up study a year later, they used high-contrast blue tableware with low-contrast (pastel) red and low-contrast blue tableware [18]. Donnelly et al. [28] compared dietary intakes between blue and white dishware at mealtimes using a menu cycle that had an increased frequency of low-contrast food offerings, such as white rice and mashed potato [28]. Hansen et al. utilised four different décor and coloured tableware at mealtimes during the testing periods [29,33]. Brush et al. enhanced visual contrast in the dining room using lighting techniques, with contrasting tablecloths and tray liners that were placed to create a contrast between the white plates [17].

### 3.6. Outcome Measures

Details of outcome measures are described in Table 3. Food intake was a primary outcome in all studies. Various strategies to determine food intake were employed in each study. Both Dunne et al. [18] and Donnelly et al. [28] used weighted measures to derive total mean percentage intakes of food intake, while Hansen et al. [29,33] and Brush et al. used photographic analysis to determine food intakes. Hansen et al. [29] reported on the percentage of individuals who fully consumed a meal based on a given plate design or colour. In addition to food intake, studies included additional outcome measures. Dunne et al. [18] also explored liquid intake, while Donnelly et al. and Hansen et al. incorporated an observational component [28,29]. Donnelly and authors [28] investigated eating challenges using standardised checklists, and Hansen et al. [29] observed mealtimes and investigated interactions with different plate colour combinations. Additionally, Hansen et al. [29] also conducted interviews with care staff to gather empirical data. The interviews were fast focus group discussions [29]. Brush et al. [17] used Mealtime Assistance Screening (MAST) and Communication Outcome Measure of Functional Independence (COMFI) tools to assess functional abilities, including psychosocial interactions; mealtime independence; cognition and communication; and mealtime behaviours, such as mealtime prerequisites, dentition, type of assistance provided, and eating problems. Hansen et al. [33] explored food waste associated with different plate colours using a quasi-experimental method, where data were extracted using an indirect approach, meaning all dishes were photographed before and after participants had eaten. The photographs were used to estimate the percentage of food consumed for each meal, and then food wasted in a single meal was subsequently calculated.

### 3.7. Food and Liquid Intake

Across all studies, increases in total food intake were seen using different coloured tableware and visual contrast enhancements. However, overall findings and statistical significance varied across studies (Table 3). Dunne et al. [18] found a significant increase in food and liquid intake with high-contrast tableware compared with white tableware, with a mean percent increase reported as 24.7% and 83.7% for food and liquid, respectively; overall, 90% of participants exhibited at least a 10% increase in food or liquid intakes during the intervention period. In comparison, Donnelly et al. [28] reported that the average number of participant observations was 19 ± 2 meals and found that only less than a third (28%) of participants increased intake by at least 10% during the intervention periods when blue tableware was used. It is difficult to explain the large variation in improved intakes between the Dunne et al. [18] and Donnelly et al. [28] studies; however, Donnelly had double the participants (*n* = 18, five males) with dementia, whereas Dunne had nine males with probable AD. Further baseline food intakes were not reported, so those with very poor starting intakes may improve more than those with near-adequate or adequate food intakes. Similarly, details of the dining environments or exact shades of the colour blue or contrast values of the studies were not reported. Further, while increased intakes observed were not statistically significant, the changes are clinically meaningful. Increasing dietary intake by 10% is important, particularly with populations at risk of malnutrition. Similarly, Hansen et al. [29] found differences in intakes with different coloured plates, where the highest food intakes were associated with the use of plates with a white well, yellow lip, and red rim—seven residents (63.4%) ate all the food on their plate; conversely, the lowest intakes were seen using standard white porcelain plates—only 36% of residents consumed all their meal. Brush et al. [17] found an increase in overall three-day calorie intake across both facilities; however, only one was statistically significant (*p* < 0.01). Facility 1 was not associated with statistically significant increases; however, an increase of over 1000 kcal in energy intake (from 3277 to 4338 kcal) was observed. On the other hand, facility 2 saw an increase from 3571 to 4475 kcal during the intervention period. Across the study, 23 out of 25 residents experienced an increase in calorie intake after enhancing table setting contrast and lighting.

### 3.8. Observations and Interviews

Care staff were interviewed in the Hansen et al. study [29]. Interviews were fast focus group discussions lasting from 20 to 45 min, and researchers conducted direct observations during mealtimes. Employees noted that coloured dishware was a refreshing alternative for dinnertime and may have enhanced the mealtime experience. It was also found that porcelain design gave residents with dementia better contrast. All coloured plates received some positive feedback from the residents, while the standard white porcelain plates did not prompt remarks during observations.

### 3.9. Eating Challenges and Mealtime Behaviours

Donnelly et al. [28] reported that wandering, using tableware improperly, and falling asleep during mealtimes were the most common eating challenges observed.

Further, Donnelly et al. [28] found that the total number of eating challenges was low (*n* = 48) and not significantly different between intervention and baseline dishware conditions. Comfort, Wearing Experience, and Function Instrument (COMFI) scores increased significantly (54 at baseline to 74 post-intervention) in the Brush et al. [17] study, associated with improvements in residents’ ability to find napkins, reduced anxiety at mealtimes, and less assistance needed (Table 3). In facility 1, MAST scores were consistent at baseline (10.7) and post-intervention (10.8, *p* = 0.977); in the other facility, MAST scores decreased but did not reach significance. These scores reportedly indicated overall improvement in functional independence at mealtimes [17].

## 4. Discussion

We sought to examine enhancing the visual contrast dining environment improved dietary intake. However, given the limited number of publications on this specific research area as well as the methodological variations, this review has identified a clear evidence gap instead of establishing the effectiveness of visual contrast interventions. We have identified key insights from the included papers, which highlight a possible relationship between visual contrast enhancements in the dining environment and food intake. It is likely that high visual contrast between food and tableware in the dining environment impacts food visibility, which may be particularly beneficial to those with visual contrast deficits and where visual contrast deficits can be common, such as in neurodegenerative diseases like dementia. However, different studies with varying interventions, including different types of contrast modification (from tableware to table settings and lighting), produced different results. Dunne et al. [18] illustrated that high-contrast dishware can significantly increase food and liquid intake in patients living with AD, suggesting that modifying visual contrast in the environment can help individuals to overcome visual deficits associated with dementia. This is supported by Brush et al. [17], who demonstrated that improved lighting and table setting contrast led to increased oral intake in aged care facilities. Similarly, Hansen et al. [29] showed coloured plates improved food intake but not when white porcelain plates were used, which suggests the material of tableware may be an important consideration. Porcelain plates that are glossy may create glare or reflect ambient light as visual clutter, creating a distraction from food intake. Non-glossy, plain, matte plates may help eliminate any distractions from food intake. Further, the authors did not report the types of food served on the plates to demonstrate contrast between food and tableware. Donnelly et al. [28] challenged this, finding no significant improvement in food intake with blue dishware compared to white in meals containing low-contrast foods, like mashed potato and white rice, indicating that colour alone may not be sufficient to improve dietary intake. Whilst some studies report small but significant increases in dietary intake, or changes in dietary intake that were not statistically different between conditions or from baseline, increases in dietary intake even by 10% is clinically meaningful given the population explored and risk of weight loss and malnutrition. An increase in dietary intake of 25% or a quarter of habitual dietary intake as seen in Dunne et al. [18] is clinically important, despite a study sample of *n* = 9. It appears that visual contrast may have a significant effect on food intake; however, the methodological weaknesses of the studies including the types of meals and food used to provide contrast for tableware and the lack of literature on this topic make it challenging to draw conclusions. Further, the methods used to capture dietary intake varied immensely from recording food intake by weighing to taking photographs to calculate food intake. The lack of unstandardised methods to capture food intake makes accurately estimating intake difficult. There is also no strong consensus on which specific colours or tableware types are most effective. This ambiguity arises from the limited number of studies and the variety of colours used, making direct comparisons challenging. Overall, the studies agree that food intake in the elderly, particularly those living in aged care or living with dementia, is influenced by external factors beyond visual contrast. A multi-interventional approach considering meal quality, accessibility, and the overall dining experience is necessary to fully address this issue.

### 4.1. Strengths

This review is the first systematic exploration of how visual contrast modifications in tableware impact food intake among aged care residents. A notable strength across the included papers is the appropriateness of their research designs in addressing their research questions and objectives. Dunne et al. [18] and Donnelly et al. [28] employed accurate and appropriate quantitative measures to assess food intakes. Dunne et al. [18] precisely measured intakes across baseline, intervention, and post-intervention, and calculated percent intakes by comparing the amount consumed with the amount served. The repeated measures enabled within-subject comparisons, and the inclusion of a follow-up replication phase strengthened the reliability of the results. Similarly, Donnelly et al. [28] used a robust crossover design to compare blue and white dishware and assessed food intake as percentages by weighing food pre- and post-mealtime, offering similar quantitative measures. The addition of additional outcome measures added to the overall findings and interpretation of results. The use of standardised checklists by Donnelly et al. [28] at mealtimes expanded the study scope beyond food intake and incorporated other aspects of the mealtime experience. Similarly, Brush et al. [17] and Hansen et al. [29] expanded the scope beyond food intake and provided insights into mealtime behaviours. Brush et al. [17] incorporated COMFI and MAST to measure changes in residents’ communication and behaviour during mealtimes. This approach allowed for a more comprehensive overview of how environmental modifications to the dining environment not only affected food intake but also residents’ overall functioning and social interactions during mealtimes. While Hansen et al.’s [29] research employed a mixed methods approach, the observational component and interview-based findings allowed for deeper exploration of the factors influencing food intake and mealtime experiences beyond visual contrast in individuals living in aged care. This review also highlighted gaps in the current literature, which should be used as the foundation to drive future research area in this field.

### 4.2. Limitations

There are several limitations to this review that should be considered. Firstly, the small number of included studies reduces their generalisability and ability to inform organisational or clinical practice. In fact, it is difficult to draw finite conclusions about relationships between visual contrast and dietary intake from these studies, having instead identified a large evidence gap. Secondly, it was not feasible to conduct a meta-analysis due to heterogeneity of methodology, small sample sizes, and differences in outcome measures; therefore, the findings remain descriptive and should be considered preliminary. Furthermore, the quality of the studies themselves was variable, affecting the overall reliability of the findings. The studies included have small sample sizes, ranging from 9 to 25 participants, limiting the statistical power and external validity of the results, which, when combined with the variability in study designs (crossover trials, mixed methods, and quasi-experimental), makes it more difficult to draw meaningful conclusions. While Donnelly et al. [28] included information on their sampling methods, this was not detailed in the other four papers. This makes it difficult to evaluate how representative the samples are of the broader population. Confounding variables were widely unaccounted for across the included studies, including cognitive status, symptoms associated with dementia, levels of assistance during mealtimes, baseline conditions of the dining environment, room layout, table setting, lighting, food type, and staff interactions, weakening the ability to solely attribute changes in food intake to the visual contrast interventions. Factors such as the room layout, table setting, and staff interactions are important to report, as these influence the social environment during mealtimes. There was largely inconsistent and inadequate reporting of various elements of the selected studies, including sample characteristics; intervention details, including meals and types of food served to create a contrast between meals and tableware; and outcome measures. This makes it more difficult to determine where bias may have been introduced and whether results were interpreted correctly. Further, the visual contrast sensitivity of the participants in the studies were not assessed, so it is difficult to determine if visual function was a factor in the outcomes of the trials. Dunne et al. [18] attempted to measure acuity colour discrimination and visual contrast; however, none of the participants could complete the assessments. Similarly, the visual contrast of the meals and tableware was not quantified, making it difficult to determine how much change in visual contrast is required to see a potential effect.

### 4.3. Implications for Future Research

This review identifies several important gaps that future research must address to advance understanding and inform practical and policy guidelines. While the studies included in this review suggest a potential relationship between visual contrast and increased food intake, the findings remain inconsistent, limiting the ability to draw conclusions and inform future policy and clinical guidelines. Although the studies included in this review have demonstrated increases in food intakes, it remains unclear whether these translate to meaningful outcomes in the long term, such as reduced prevalence of malnutrition, reduced unintentional weight loss, or optimised nutritional status among older adults in long-term aged care. The current evidence base included in this review focuses exclusively on individuals with AD or dementia, leaving a significant gap in our understanding of how visual contrast might affect older adults in different care settings, such as within the community or in acute settings, including hospital settings, and whether similar strategies involving enhancing visual contrast in the dining environment would benefit cognitively healthy individuals. Future research should therefore aim to conduct larger, well-powered studies with standardised methodologies and longer intervention periods. They should quantify colour–contrast ratios and investigate a wider range of visual contrast interventions, such as different colours and patterns, which may help identify more specific and effective approaches to support food intake. Further, future research should focus on the precise values of brightness and colour that are needed to overcome visual sensitivity deficits, as well as the visual contrast of the meal and tableware, when designing interventions, with the important assessment of visual contrast sensitivity of the target population. Failing to account for any of these considerations may compromise the validity of the results. This is a significant limitation in the studies reviewed. To establish evidence-based guidelines, future research should also consider the broader mealtime context, including meal quality, other aspects of the dining environment, and meal provision alongside individual preferences and needs, as well as functional and clinical assessments related to weight loss and malnutrition, such as change in weight and subcutaneous fat scores, biochemical markers (e.g., serum albumin), and malnutrition screening tools like the Mini Nutritional Assessment (MNA). Addressing these factors will support the development of specific and effective strategies to optimise nutritional intake in diverse aged care populations.

## 5. Conclusions

This review has identified a clear evidence gap in research exploring visual contrast and dietary intake. The preliminary nature of the available pioneering pilot studies, albeit with low MMAT scores, provide some evidence that increasing visual contrast in the dining environment encourages increased food intake among residents living in aged care facilities, particularly those with cognitive impairments. However, due to methodological limitations of the included studies, including different visual contrast environments and outcome measures, there remains a significant knowledge gap, and the evidence remains inconclusive. More robust, well-designed studies with larger population sizes, homogenous interventions, longer study periods, better control of confounding factors, and improved reporting standards are warranted to clarify the role of visual contrast in enhancing food intake. Future research in this area should establish whether these simple interventions can have long-term improvements in nutritional status. These interventions, if proven effective, have the potential to be a cost-effective strategy to address malnutrition in aged care settings and inform future clinical practice and policy guidelines.

## Figures and Tables

**Table 1:** Study characteristics.

Author, Year	Study Design & Duration	Sample Size (Male/Female)	Age (As Reported)	Setting	Cognitive Status	Intervention	Outcome Measures
Donnelly et al., 2020 [28]	Crossover trial. Lunch and dinner observed 4 days in weeks 3 & 4 of a 4-week menu cycle.	18 (5/13)	84.6 ± 7.9 years *	Memory care unit within retirement home, Canada	Dementia	Blue dishware compared to white dishware. Lunches and dinners served with frequent low-contrast foods, e.g., mashed potato and white rice.	Food intake & eating challenges
Hansen et al., 2018 & 2020 [29,33]	Mixed methods & quasi-experimental. Three-week intervention with 4 days randomly selected.	12 (7/5)	65–85 years	Two separate units in a nursing home, Norway	Physical diagnosis of dementia	Different décor and dinner plate colours. Four porcelain plates: white; yellow well and red lip; white well, green lip, and blue rim; and white well, yellow lip, and red rim. Dinner served, but type of food not reported.	Food intake, appetite, mealtime behaviours & food waste
Dunne et al., 2004 [18]	Quasi-experimental. Data collected for 3 consecutive 10-day periods days (10 days per condition, 2 meals per day). Follow-up study done 1 year later (3 × 10-day periods as protocol above).	9 (9/0)	72–89 years	Long-term care setting, Massachusetts, USA	Probable Alzheimer’s Disease	Baseline white tableware vs. red tableware (cups, plates, flatware). Meals included lunch and supper with low-contrast foods, including chicken, mashed potatoes, and milk. Follow-up study 1 year later using the same study design, with high-contrast blue, high-contrast red tableware, and low-contrast (pastel) red and low-contrast blue tableware.	Food & liquid intake
Brush et al., 2002 [17]	Quantitative non-randomised pilot study. Data collected at baseline and at week 4.	25 (3/22)	≥70 years	One residential & one assisted living facility in a long-term care home, Wisconsin, USA	Any type of dementia	Lighting—light levels measured in foot-candles at the table surface (task lighting), with light level readings used to determine contrast ratio of lighting in the dining room.	Food intake, mealtime experience & functional abilities

* mean ± SD.

**Table 2:** Results of quality assessments based on the Mixed Methods Appraisal Tool^51^ for qualitative, quantitative, and mixed methods reports.

	Quantitative Non-Randomised				Mixed Methods
	Dunne et al., 2004 [18]	Donnelly et al., 2020 [28]	Hansen et al., 2020 [33]	Brush et al., 2002 [17]	Hansen et al., 2018 [29]
Screening Questions:					
S1. Are there clear research questions?	Y	Y	Y	Y	Y
S2. Do the collected data address the research questions?	Y	Y	Y	Y	Y
Quality Criteria:					
1.1. Is the qualitative approach appropriate to answer the research questions?					
1.2. Are the qualitative data collection methods adequate to address the research questions?					
1.3. Are the findings adequately derived from the data?					
1.4. Is the interpretation of results sufficiently substantiated by the data?					
1.5. Is there coherence between qualitative data sources, collection, analysis, and interpretation?					
Randomised Controlled Trials:					
2.1. Is randomisation appropriately performed?					
2.2. Are the groups comparable at baseline?					
2.3. Are there complete outcome data?					
2.4. Are outcome assessors blinded to the intervention provided?					
2.5. Did the participants adhere to the assigned intervention?					
Non-Randomised Studies:					
3.1. Are the participants representative of the target population?	N	CT	CT	N	
3.2. Are measurements appropriate regarding both the outcome and intervention (or exposure)?	Y	Y	Y	Y	
3.3. Are there complete outcome data?	CT	CT	CT	CT	
3.4. Are the confounders accounted for in the design and analysis?	N	N	CT	CT	
3.5. During the study period, is the intervention administered (or exposure occurred) as intended?	Y	Y	Y	Y	
Quantitative Descriptive Studies:					
4.1. Is the sampling strategy relevant to address the research questions?					
4.2. Is the sample representative of the target population?					
4.3. Are the measurements appropriate?					
4.4. Is the risk of non-response bias low?					
4.5. Is the statistical analysis appropriate to answer the research questions?					
Mixed Methods Studies:					
5.1. Is there an adequate rationale for using a mixed methods design to address the research questions?					Y
5.2. Are the different components of the study effectively integrated to answer the research questions?					Y
5.3. Are the outputs of the integration of qualitative and quantitative components adequately interpreted?					Y
5.4. Are divergences and inconsistencies between quantitative and qualitative results adequately addressed?					CT
5.5. Do the different components of the study adhere to the quality criteria of each tradition of the methods involved?					CT
Score	40%	40%	40%	40%	60%

Y = yes, N = no, CT = cannot tell. Score: The overall score ranges from 0% (if no quality criteria is met) to 100% (if all five quality criteria are met). For mixed methods studies including 15 criteria, the overall quality score is the lowest score of the study components.

**Table 3:** Key findings by outcome measure.

Outcome Measure	Author	Intervention	Result	Statistical Significance
Food Intake	Donnelly et al. [28]	Blue dishware compared to white dishware. Lunches and dinners served with frequent low-contrast foods, e.g., mashed potato and white rice.	Average mean difference was 4.8 ± 7% with blue dishware.	No difference between dishware—*p* > 0.05
	Dunne et al. [18]	Baseline white tableware vs. red tableware (cups, plates, flatware). Meals included lunch and supper with low-contrast foods, including chicken, mashed potatoes, and milk.	Initial study:	
			High-contrast red tableware: 24.6% increase in food intake compared with white.	*p* < 0.01
			Food intake different between baseline (white), intervention (red), and post-intervention (flatware).	*p* = 0.001
		Follow-up study 1 year later using the same study design, with high-contrast blue, high-contrast red tableware, and low-contrast (pastel) red and low-contrast blue tableware.	Follow up study:	
			High-contrast blue: mean percent increase of 25.1%.	Significant, but *p* values not provided
			No increase in low-contrast red.	Low-contrast intervention not significant
			Low-contrast blue: 5.2% average increase.	
	Brush et al. [17]	Lighting—light levels measured in foot-candles at the table surface (task lighting), and light level readings were used to determine contrast ratio of lighting in the dining room. Types of food served were not reported.	Facility 1:	
			>1000 kcal increase in the average 3-day calorie count.	*p* < 0.16
			Facility 2:	
			Significant increase (3571 to 4475 kcal) in average total kcals consumed.	*p* < 0.01
	Hansen et al. [33]	Different décor and dinner plate colours. Four porcelain plates: white; yellow well and red lip; white well, green lip, and blue rim; and white well, yellow lip, and red rim. Dinners served, but types of food were not reported.	Food intake varied by plate design: 36% of residents ate all food on a white plate; 60% on a yellow well and red lip plate; 42% on a white well with green lip and blue rim; and 63.4% on a white well with a yellow lip and red rim, which was associated with the highest intakes.	Small sample size did not allow for statistical testing
Liquid Intake	Dunne et al. [18]	“High-contrast” red and blue versus “low-contrast” white, pastel red, and blue tableware. Meals included lunch and supper with low-contrast liquids, e.g., milk.	Initial study:	
			Increase of 83.7% with high-contrast red tableware.	*p* = 0.001
			Follow-up study:	
			Increase of 29.8% with high-contrast blue	No *p* value provided
			Increase of 0.4% with low-contrast red	No *p* value provided
			Increase of 0.3% with low-contrast blue	No *p* value provided
Mealtime Behaviours & Eating Challenges	Donnelly et al. [28]	Blue dishware compared to white dishware. Lunches and dinners served with frequent low-contrast foods, e.g., mashed potato and white rice.	Proportion of eating challenges was not significantly different between blue (14%) and white tableware (9.4%) conditions.	*p* = 0.17
	Hansen et al. [29]	Different décor and dinner plate colours. Four porcelain plates: white; yellow well and red lip; white well, green lip, and blue rim; and white well, yellow lip, and red rim. Dinners served, but types of food were not reported.	All new colour combinations got positive comments from some of the residents, and colour may have enhanced the mealtime experience; the porcelain design might give residents with dementia better contrast between the plate and the table.	Not assessed
Functional Abilities	Brush et al. [17]	Lighting—light levels measured in foot-candles at the table surface (task lighting), and light level readings were used to determine contrast ratio of lighting in the dining room.	Facility 1:	
COMFI *			Significant increase in COMFI scores (54.0 to 72.09).	*p* = 0.018
MAST **			Residents’ ability to find and use their napkins improved.	*p* < 0.16
			Increased frequency of residents engaging in conversation with staff members.	*p* < 0.05
			Increased frequency of residents starting conversations with staff.	*p* < 0.01
			No change in MAST scores.	*p* = 0.977
			Facility 2:	
			Significant increase in COMFI scores (48.28 to 60.71).	*p* = 0.115
			Reduction in residents’ anxiety.	*p* < 0.01
			Reduction in assistance required.	*p* < 0.01
			Improved ability to follow simple directions at mealtimes.	*p* < 0.01
			Decrease in MAST scores (baseline 6.21, post-intervention 4.86)	*p* = 0.331
			Significant reduction in distractibility.	*p* < 0.05
Food Waste	Hansen et al. [33]	Different décor and dinner plate colours. Four porcelain plates: white; yellow well and red lip; white well, green lip, and blue rim; and white well, yellow lip, and red rim. Dinners served, but types of food were not reported.	On average, 26% of food was thrown away when served on white plates compared to only 9% when served on one of the coloured plate options. It was calculated that approximately 992.6 tonnes of food could be saved annually with a single change when the results of the pilot study were extrapolated to annual food waste at national level in Norway.	Not assessed

* Communication Outcome Measure of Functional Independence (COMFI) provides a measure of how an individual functions and communicates within the long-term care setting. The COMFI scale includes 20 items that measure performance in the following four areas: psychosocial interaction, communication and conversation, mealtime independence, and cognition. Items are given a score between zero and five, and subjects receive a total score between zero and 100. A higher score indicates higher functioning. Scores were based on what was observed during nine consecutive meals. ** Mealtime Assistance Screening Tool (MAST) examines the following eight areas related to mealtime: mealtime prerequisites, seating/positioning problems, dentition and oral hygiene, type of diet provided, type of assistance provided, intake, challenging behaviours, and eating problems.

## Data Availability

No new data were created or analyzed in this study. Data sharing is not applicable to this article.

## Supplementary Materials

The following supporting information can be downloaded at: https://www.mdpi.com/article/10.3390/nu18142338/s1, Table S1: MEDLINE Search Strategy.

## Abbreviations

The following abbreviations are used in this manuscript:

AD	Alzheimer’s Disease
RCT	Randomised Controlled Trial
MMAT	Mixed Methods Appraisal Tool
MoCA	Montreal Cognitive Assessment
MMSE	Mini-Mental State Exam
MDS	Minimum Data Set
MAST	Mealtime Assistance Screening Tool
COMFI	Communication Outcome Measure of Functional Independence
