# Supplementary material for: Modification of Visual Contrast in the Dining Environment and Its Impact on Dietary Intake in Older Adults—A Systematic Review
Source: Nutrients. 2026 Jul 16;18(14):2338. doi: 10.3390/nu18142338 (PMC13415140; doi:10.3390/nu18142338)
Supplement: Supplementary file 1 [file nutrients-18-02338-s001.zip › nutrients-4385468-supplementary.pdf]

Supplementary Table S1. MEDLINE Search Strategy.

Search Category	Search Terms
Aged	"aged, 80 and over" OR “oldest old’ OR “aged’ OR “elderly”
Aged Care	“homes, nursing” OR “nursing homes” OR “age friendly health care" OR “age friendly health services" OR “age friendly health systems" OR “age-friendly health cares" OR “age-friendly health service" OR “age-friendly health system" OR “care, age-friendly health" OR “care, geriatric health" OR “cares, age-friendly health" OR “cares, geriatric health" OR “geriatric health care" OR “geriatric health cares" OR “geriatric health service" OR “geriatric health services" OR “geriatric health system" OR “geriatric health systems" OR “health care, age-friendly" OR “health care, geriatric" OR “health care for the aged" OR “health cares, age-friendly" OR “health cares, geriatric" OR “health service, age-friendly" OR “health service, geriatric" OR “health services, age-friendly" OR “health services, geriatric" OR “health services for aged" OR “health services for the aged" OR “health services for the elderly" OR “health system, age-friendly" OR “health system, geriatric" OR “health systems, age-friendly" OR “health systems, geriatric" OR “health systems for the aged" OR “service, age-friendly health" OR “service, geriatric health" OR “services, age-friendly health" OR “services, geriatric health" OR “system, age-friendly health" OR “system, geriatric health" OR “systems, age-friendly health" OR “systems, geriatric health” OR “home, old age" OR “homes, old age" OR “homes for the aged" OR “housing, senior" OR “old age home" OR “old age homes" OR “residential aged care facility" OR “senior housing” OR “hospital" OR “hospitals”
Cognitive Dysfunction	“cognitive decline” OR “cognitive declines” OR “cognitive disorder” OR “cognitive disorders” OR “cognitive dysfunction” OR “cognitive dysfunctions” OR “cognitive impairment” OR “cognitive impairment, mild” OR “cognitive impairments” OR “cognitive impairments, mild” OR “decline, cognitive” OR “declines, cognitive” OR “deterioration, mental” OR “deteriorations, mental” OR “disorder, cognitive” OR “disorders, cognitive” OR “dysfunction, cognitive” OR “dysfunctions, cognitive” OR “impairment, cognitive” OR “impairment, mild cognitive” OR “impairments, cognitive” OR “impairments, mild cognitive” OR “mental deterioration” OR “mental deteriorations” OR “mild cognitive impairment” OR “mild cognitive impairments”
Dementia	“amentias" OR “dementia" OR “dementia, familial" OR “dementias" OR “dementias, familial" OR “dementias, senile paranoid" OR “familial dementia" OR “familial dementias" OR “paranoid dementia, senile" OR “paranoid dementias, senile" OR “senile paranoid dementia" OR “senile paranoid dementias”
Alzheimer Disease	“acute confusional senile dementia" OR “alzheimer dementia" OR “alzheimer dementias" OR “alzheimer disease" OR “alzheimer disease, early onset" OR “alzheimer disease, familial (fad) " OR “alzheimer disease, late onset" OR “alzheimer diseases" OR “alzheimer sclerosis" OR “alzheimer syndrome" OR “alzheimer type dementia" OR “alzheimer type dementia (atd) " OR “alzheimer type senile dementia" OR “alzheimer's disease" OR “alzheimer's disease, focal onset" OR “alzheimer's diseases" OR “alzheimers diseases" OR “dementia, alzheimer" OR “dementia, alzheimer type" OR “dementia, alzheimer-type (atd) " OR “dementia, presenile" OR “dementia, primary senile degenerative" OR “dementia, senile" OR “early onset alzheimer disease" OR “familial alzheimer disease (fad) " OR “familial alzheimer diseases (fad) " OR “focal onset alzheimer's disease" OR “late onset alzheimer disease" OR “presenile alzheimer dementia" OR “presenile dementia" OR “primary senile

	degenerative dementia" OR “sclerosis, alzheimer" OR “senile dementia" OR “senile dementia, acute confusional" OR “senile dementia, alzheimer type”
Visual Contrast	Contrast Sensitivity:<br>“contrast sensitivity" OR “sensitivity, contrast" OR “sensitivity, visual contrast" OR “visual contrast sensitivity”<br><br>Visual Acuity:<br>“acuities, visual" OR “acuity, visual" OR “visual acuities" OR “visual acuity”
Colour	Colour:<br>“color" OR “colors”<br><br>Colour Perception:<br>“color perception" OR “color perceptions" OR “perception, color" OR “perceptions, color”
Cooking and Eating Utensils	“cooking utensil" OR “cooking utensils" OR “cooking and eating utensils" OR “eating utensil" OR “eating utensils" OR “utensil, cooking" OR “utensil, eating" OR “utensils, cooking" OR “utensils, eating”
Nutrition	“nutrition”<br><br>Meals:<br>“dinner" OR “dinner time" OR “dinner times" OR “dinners" OR “meal" OR “meal time" OR “meal times" OR “meals" OR “supper" OR “suppers" OR “time, dinner" OR “time, meal" OR “times, dinner" OR “times, meal”<br><br>Eating:<br>“dietary intake" OR “dietary intakes" OR “eating" OR “feed intake" OR “feed intakes" OR “food intake" OR “ingestion" OR “intake, dietary" OR “intake, feed" OR “intake, food" OR “intake, macronutrient" OR “intake, micronutrient" OR “intake, nutrient" OR “intake, nutritional" OR “macronutrient intake" OR “macronutrient intakes" OR “micronutrient intake" OR “micronutrient intakes" OR “nutrient intake" OR “nutrient intakes" OR “nutritional intake" OR “nutritional intakes”
Weight Loss	Weight Loss:<br>“loss, weight" OR “losses, weight" OR “reduction, weight" OR “reductions, weight" OR “weight loss" OR “weight losses" OR “weight reduction" OR “weight reductions”<br><br>Malnutrition:<br>“malnourishment" OR “malnourishments" OR “malnutrition" OR “nutritional deficiencies" OR “nutritional deficiency" OR “undernutrition”
